# Novel Use of Lysis-Assisted Covered Endovascular Reconstruction of the Aortic Bifurcation (LA CERAB) in Acute Aortobiiliac Graft Thrombosis: A Case Report

**DOI:** 10.1177/15385744251409967

**Published:** 2025-12-23

**Authors:** Ajibola O. Anifowose, Aneet Mann, Harneil Sidhu, Matt Cwinn

**Affiliations:** 1 Faculty of Medicine and Dentistry, 3158 University of Alberta, Edmonton, AB, Canada; 2 Division of Vascular Surgery, 3158 University of Alberta, Edmonton, AB, Canada; 3 Division of Interventional Radiology, 3158 University of Alberta, Edmonton, AB, Canada

**Keywords:** aortoiliac occlusive disease, aortobifemoral bypass, covered endovascular reconstruction of the aortic bifurcation (CERAB), endovascular procedures, peripheral arterial disease, aortoiliac graft thrombosis, claudication, critical limb ischemia, vascular surgical procedures, revascularization

## Abstract

**Introduction:**

Acute aortic occlusion (AAO) is a rare, life-threatening condition presenting with severe ischemia and requiring urgent intervention. While traditional open surgical approaches, including aortobifemoral and axillobifemoral bypasses, are well-established, endovascular techniques such as Covered Endovascular Reconstruction of the Aortic Bifurcation (CERAB) have emerged as promising alternatives in select cases. This case introduces a novel modification termed Lysis Assisted CERAB (LA CERAB), combining thrombolysis and CERAB to manage acute graft occlusions.

**Methods:**

Single-institution case report highlighting the utility of the LA CERAB technique in select patients.

**Results:**

A 67-year-old male presented two years post-open aortobiiliac aneurysm repair with acute abdominal pain and bilateral limb ischemia without motor impairment. Initial CTA demonstrated complete thrombosis of his graft. He underwent percutaneous bilateral transfemoral catheter-directed thrombolysis. Post-thrombolysis angiography showed graft patency but significant residual thrombus. Subsequent LA CERAB successfully re-lined the graft, secured residual thrombus, and restored perfusion. The patient had an uneventful recovery, discharged home on therapeutic anticoagulation. Follow-up CTA at 2 months demonstrated sustained graft patency. This case highlights its applicability to acute presentations and adds to a growing body of literature on acute aortic graft occlusion interventions.

**Conclusion:**

The LA CERAB technique can serve as a novel, minimally invasive approach for select AAO patients presenting with high morbidity risks for transition open surgery. This case underscores patient-specific considerations in treatment strategy selection, illustrating how individualized surgical approaches can achieve favorable immediate and sustained clinical outcomes for future patients.

## Introduction

Acute aortic occlusion (AAO) is a rare clinical presentation that often has devastating consequences. Patients often present with signs and symptoms of advanced ischemia, and mortality rates up to 23% have been reported.^1^ Open surgical management of AAO includes aortobifemoral bypass and axillobifemoral bypass.^1,2^ Recently, a small series describing endovascular management of AAO has been published.^3^ However, given the rarity of the presentation and the heterogeneous nature in which patients may present, the ideal surgical approach will depend on patient presentation, comorbidities, surgeon experience, and local resources.

In contrast, aortoiliac occlusive disease (AIOD) is a much more common vascular condition characterized by progressive occlusion of the infrarenal aorta and iliac arteries. AIOD is prevalent in older populations, with estimates suggesting it affects 15-25% of individuals above 70 years old.^4^ Symptoms of AIOD can range from being asymptomatic to presenting with chronic limb-threatening ischemia. However, these estimates may be underestimated as current data often do not account for asymptomatic patients who may have undiagnosed AIOD.^5-8^

The ideal approach to AIOD whether open, endovascular, or hybrid, depends on a variety of factors including patient comorbidities, symptoms, and anatomy. Covered endovascular reconstruction of the aortic bifurcation (CERAB) is a relatively novel surgical technique to treat aortoiliac occlusive disease.^9^ The CERAB technique is an endovascular procedure that entails the use of a covered stent graft for the infrarenal portion of the aorta and then two stent-grafts deployed within the infrarenal aortic stent to extend into both common iliac arteries, thus creating an endovascular neo-bifurcation.^10^ CERAB offers favorable flow dynamics for patients with significant occlusion in the distal aorta and may be advantageous for those with significant circumferential calcification of the distal aorta and common iliac arteries.^10^ The use of thrombolysis, followed by CERAB (LA CERAB) for chronically occluded aortic pathology has been described.^11^ Importantly, there is a scarcity of data on the effectiveness of CERAB in the management of acute aortic pathology. This case provides novel evidence that LA CERAB can also be used to treat AOO in select patients.

## Case Report

A 67-year-old male initially presented with a 6.7 cm juxtarenal abdominal aortic aneurysm (AAA). His history is relevant for coronary artery disease, dyslipidemia, and a remote smoking history. The patient underwent open aortobiiliac aneurysm repair in 2022 using a 16 mm × 8 mm Dacron graft. He had an uneventful recovery and was discharged home. A routine CT scan performed in the immediate postoperative period demonstrated a satisfactory repair with a small, non-flow-limiting dissection at the right common iliac anastomosis.

**Figure 1.**
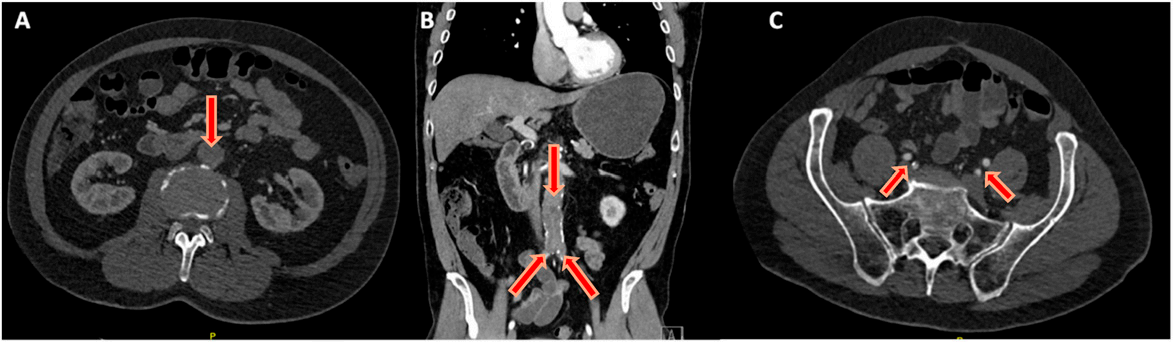
Representative cuts from a CT angiogram demonstrating complete occlusion of the aortobiiliac graft. (A) Axial image demonstrating a lack of contrast within the infrarenal aorta. (B) Coronal view demonstrating opacification of the ascending aorta (AA), Superior mesenteric artery (SMA) and left renal artery (LRA). Note the lack of contrast within the infrarenal graft. (C) Axial view demonstrating reconstitution of luminal flow in the external and internal iliac arteries via collaterals.

**Figure 2.**
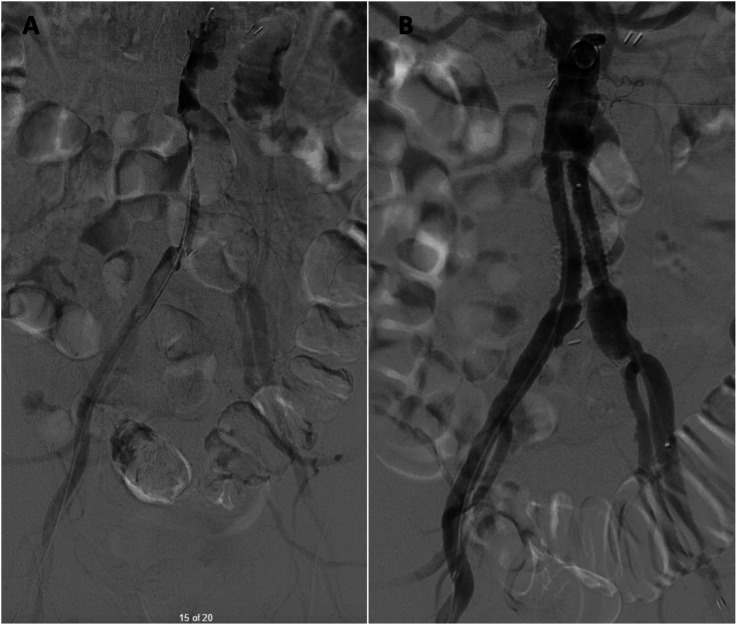
Conventional angiography before and after catheter directed thrombolysis of the occluded aortobiiliac graft. (A) Angiography demonstrating complete thrombosis of the aortobiiliac graft. (B) Angiography after 24 hours of catheter directed thrombolysis demonstrating resolution of patency.

**Figure 3.**
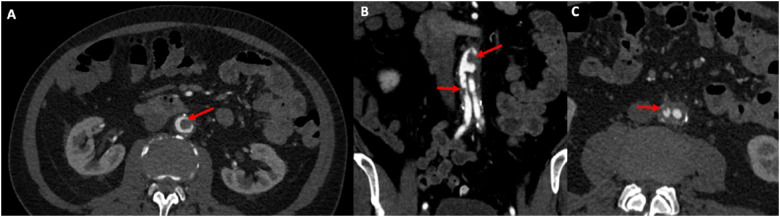
CTA post thrombolysis demonstrates a significant burden of residual thrombus within the graft (red arrows) in the aortic segment (A/B) and the right iliac limb (C).

Two years post-procedure, the patient presented to the emergency department with acute abdominal pain and bilateral limb ischemia with significant pain, paresthesia and initial lower extremity weakness and discoordination. The patient presented with Rutherford class IIa acute limb ischemia, characterized by lower-extremity pain and mild sensory deficits while preserving motor function.^12^ He had no prior history of claudication or chronic limb-threatening ischemia after his index procedure and remained asymptomatic until the onset of the acute event. Urgent CTA demonstrated complete thrombosis of his aortobiiliac graft (Figure 1). Shortly after presentation and initiation of therapeutic systemic intravenous heparin, his motor strength and coordination returned to his lower extremities. Given that his motor function was intact, the decision was made to attempt endovascular rescue of the thrombosed graft. He underwent thrombolysis via bilateral percutaneous transfemoral access. The occluded graft was crossed in a retrograde fashion. Perfusion catheters were placed, and lysis was performed per institutional protocol (Figure 2A). Briefly, a 2 mg bolus of tissue plasminogen activator (TPA) was administered through each perfusion catheter, followed by a 0.5 mg/hr infusion of TPA for 24 hours. The patient’s symptoms improved and repeated angiography after 24 hours demonstrated a patent aortobiiliac graft (Figure 2B). Repeat CTA demonstrated that despite a patent-appearing graft on conventional angiography, there was still a significant burden of thrombus within the graft. Notably, an obvious cause of graft thrombosis was not apparent (Figure 3). Therefore, a decision was made to re-line the aortobiiliac graft using the CERAB technique to trap residual aortic thrombus, prevent embolization or re-thrombosis, and treat any occult pathology leading to graft failure.

**Figure 4.**
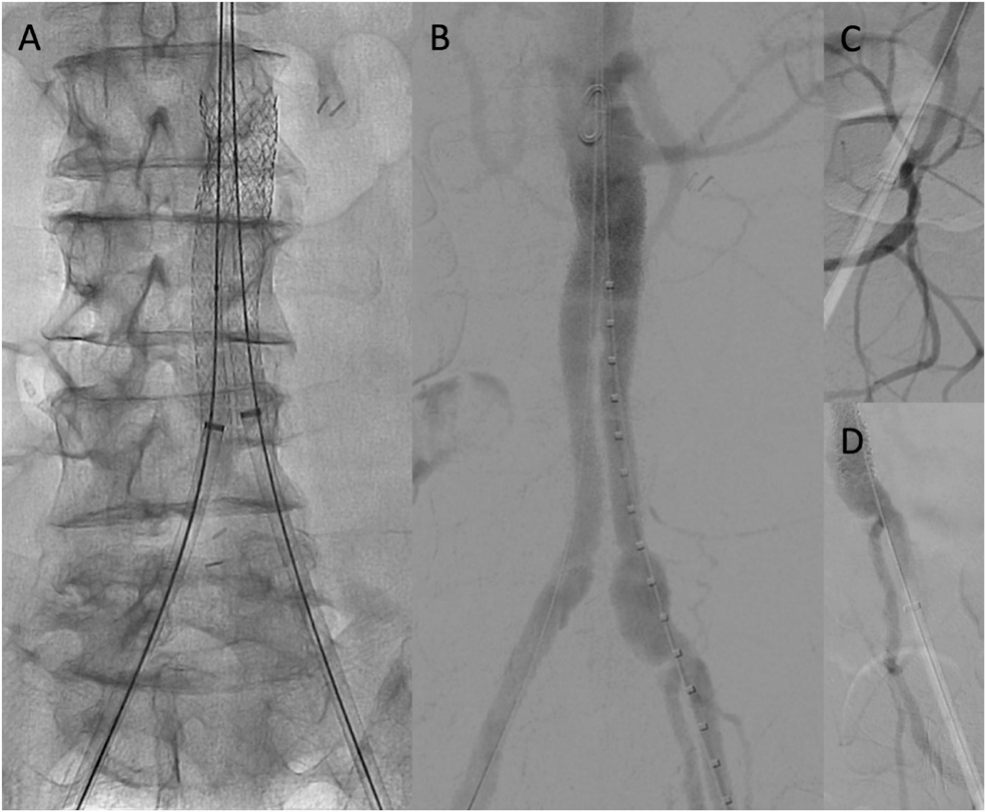
Intraoperative images demonstrating complete re-lining of the aortobiiliac graft utilizing the CERAB technique. (A) High-resolution fluoroscopy demonstrating stent graft architecture and creation of the neo-aortoiliac bifurcation within the prior aortobiiliac graft. (B) completion angiography post CERAB. Note that the right renal is significantly higher than the left. (C, D) Retrograde angiography demonstrating preserved patency of the right (C) and left (D) internal iliac arteries.

Percutaneous bilateral common femoral access was obtained. A 22 mm × 37 mm Bentley Aortic Begraft (Bentley Innomed, Germany) was deployed within the proximal aspect of the previous aortobiiliac graft below his lowest renal artery. Following this, endovascular iliac reconstruction was performed. The neo-aortic bifurcation was created using 8 mm × 79 mm Gore VBX stents (W.L. Gore & Associates, USA) and then molded with 12 mm × 40 mm Mustang balloons to post-dilate the main body as well as the limbs, recreating a neo-aortoiliac bifurcation within the previously placed graft. The limbs were then extended with a 9 mm × 57 mm Bentley Begraft Peripheral on the right and a 10 mm × 57 mm Bentley Begraft Peripheral on the left to ensure that the entire Dacron graft was now covered and that the stent grafts were sealing in the patient’s native vessels. The use of different stent graft manufacturers was due to our on-the-shelf availability given the urgency of the case. Completion angiography confirmed successful re-lining of the Dacron graft with patency of the renal arteries and bilateral internal iliac arteries (Figure 4).

**Figure 5.**
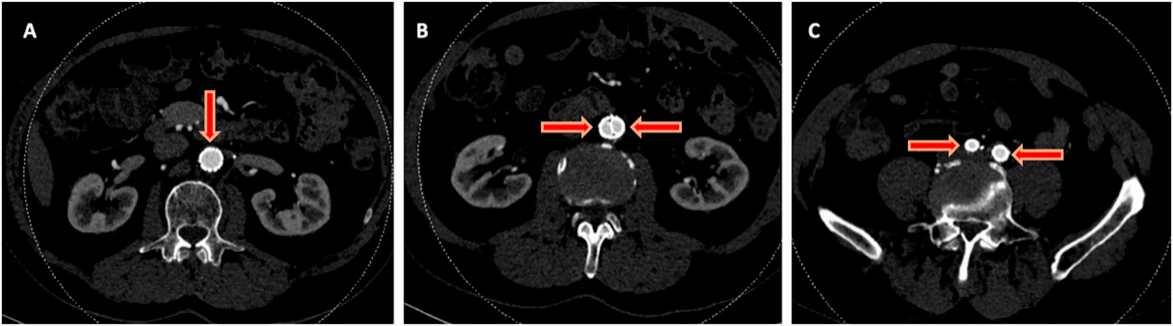
Post-operative images demonstrating a widely patent stent graft post-CERAB, with an absence of thrombus within the aortoiliac segment and an absence of iliac pathology. (A–C) represent axial images of the aorta (A), iliac bifurcation (B) and common iliac arteries (C).

The patient had an uneventful postoperative course. He was discharged home without any postoperative concerns on 81 mg of aspirin (ASA) and therapeutic anticoagulation with apixaban. At the time of discharge, rivaroxaban was not covered by the provincial pharmaceutical plan. Therefore, the COMPASS treatment protocol, which includes ASA and low-dose rivaroxaban, was not selected due to financial constraints.^13^ Follow-up CTA at 2 months demonstrated a widely patent graft with no evidence of residual thrombus (Figure 5). Ongoing patency, absence of restenosis and a stable ankle-brachial index were confirmed by duplex at 6 and 12 months (not shown).

## Discussion

The Covered Endovascular Reconstruction of the Aortic Bifurcation (CERAB) technique was introduced to improve clinical outcomes and results of endovascular treatment for chronic occlusive disease by optimizing the anatomical reconstruction.^10,14^

Numerous studies have provided clinical and empirical data to validate the efficacy of the CERAB technique in treating chronic aortoiliac occlusions. In 2023, a single-center study assessing long-term outcomes of CERAB for AIOD in 160 patients concluded that at 5-year follow-up, clinical improvement was found in 97.9% of patients without major amputations. Their 5-year overall primary, primary-assisted, and secondary patency rates were 77.5%, 88.1%, and 95.0%, respectively.^15^ Relative to the gold standard aortobifemoral bypass (ABF) for AIOD treatment, endovascular techniques using CERAB offer lower rates of morbidity and mortality, minimally invasive procedures and shorter lengths of stay.^16,17^ Similarly, a retrospective analysis of patients who underwent CERAB with anatomically complex AIOD lesions between 2012 to 2020 in six centers across the United Kingdom concluded that CERAB is a viable revascularization option. The study reported a 0% 30-day mortality rate, with 1-year primary patency, assisted primary patency, and secondary patency rates being 88%, 94%, and 98%, respectively.^18^

While CERAB has been researched and validated as a viable treatment for aortic stenosis and chronic iliac occlusive disease, patients with chronic aortic occlusions present a particularly challenging cohort of patients, and the use of CERAB in this population is less clear. A retrospective case review conducted by Minion in 2023 on six patients with chronic aortic occlusions treated with catheter-directed thrombolysis in conjunction with CERAB (Lysis-assisted CERAB - LA CERAB) has provided evidence that this may be a viable technique.^11^ Our case provides evidence that LA CERAB can also be safely and effectively used to treat acute aortic occlusion.

A unique aspect of this case is the patient presentation. Patients with AAO often present in a catastrophic fashion, including severe lower limb ischemia, paralysis, and colonic ischemia.^19,20^ These patients require emergent surgical intervention and may not be able to tolerate a period of thrombolysis. The patient’s presentation of paresthesia without persistent motor dysfunction was atypical for acute aortic occlusion (AAO), prompting consideration of alternative etiologies. AAO is commonly caused by in situ thrombosis of diseased vessels, embolic events, graft thrombosis, or arterial dissection.^21^ In this case, a limited right common iliac artery dissection was identified on postoperative CT imaging during his initial presentation. Despite this finding, the patient had a palpable femoral pulse and no signs of claudication or chronic limb-threatening ischemia. Therefore, the clinical team pursued expectant management and initiated antiplatelet therapy with aspirin (ASA) after his initial presentation.

At his second presentation, the atypical symptoms and milder severity compared to typical AAO created a clinical safety window in which LA CERAB could be considered. A range of revascularization strategies were evaluated, including thrombolysis, thrombectomy, and open surgical revascularization. Catheter directed thrombolysis carries a risk of major bleeding, including intracranial hemorrhage, and distal embolization.^22^ Furthermore, thrombolysis requires time, making it unsuitable in patients with imminent threat of limb-loss. However, in this patient, there were no contraindications to thrombolysis, and his ischemic status was stable enough that the time required for thrombolytic therapy was unlikely to compromise his chances of limb salvage.

Open transfemoral embolectomy was deemed to have a high risk of failure due to the extensive thrombus burden, and potential for embolization to the renal arteries or distal vasculature. Although endovascular thrombectomy using devices such as the AngioJet system is well described in the literature, these devices were not available at our institution.^23^ Open revascularization options, including redo aortobi-iliac reconstruction and axillobifemoral bypass were also considered. Given the patient’s young age and absence of significant comorbidity, he was likely to tolerate redo open aortic surgery. Importantly, there is considerable morbidity and mortality associated with redo open aortic surgery.^24^ Because the patient lacked significant sensory or motor deficits, the clinical team judged a minimally invasive approach to be appropriate as an initial strategy, reserving open aortic surgery for salvage if needed. Axillobifemoral bypass, known for poor long-term patency and higher rates of adverse limb events was considered a last resort.^25^ Collectively, these considerations supported the decision to proceed with LA CERAB as a minimally invasive revascularization option in this relatively young and otherwise healthy patient.

In vascular surgery, the progressive complexity of both the patient population and the surgical interventions performed creates challenging clinical scenarios. There is a need to expand the application of our interventions to provide the best possible outcomes for future patients. The LA CERAB technique serves as a novel solution for managing acute occlusion of the infrarenal aorta in high-risk patients with complex vascular anatomy and pre-existing comorbidities.

## Conclusion

This case report demonstrates the novel and successful application of the LA CERAB technique. By combining thrombolysis with CERAB, the approach not only restored perfusion but also mitigated the risk of re-thrombosis through endovascular neo-aortoiliac reconstruction. The patient’s presentation was pivotal in allowing time for an endovascular intervention, demonstrating that patient selection is critical in determining optimal management strategies. The technique presents a viable alternative to traditional open surgery, particularly in patients with significant comorbidities or when less invasive options are preferable. While outcomes in this case were favorable, the lack of robust data on the long-term durability and broader applicability of LA CERAB warrants further investigation. As the field of vascular surgery evolves and patient presentations remain diverse, integrating novel techniques like the LA CERAB can expand the scope of endovascular interventions, offering improved personalized care and outcomes for high-risk patients.

## Data Availability

All relevant data and images supporting the findings of this case report are included within the manuscript. Additional data requests may be directed to the corresponding author*.
